# Dataset of experimental study investigation of airflow and heat transfer in an insulated box equipped with a phase change material

**DOI:** 10.1016/j.dib.2022.108696

**Published:** 2022-10-26

**Authors:** Tanathep Leungtongkum, Onrawee Laguerre, Denis Flick, Alain Denis, Steven Duret, Nattawut Chaomuang

**Affiliations:** a Université Paris-Saclay, INRAE, FRISE, 92761, Antony, France; b Université Paris-Saclay, INRAE, AgroParisTech, UMR SayFood, 91120 Palaiseau, France; c Department of Food Engineering, School of Engineering, King Mongkut's Institute of Technology Ladkrabang, Bangkok, Thailand 10520

**Keywords:** Food cold chain, Heat transfer, Airflow, Insulated box, Phase change material

## Abstract

This article contains a detailed description of the experimental protocol of air velocity (by particle image velocimetry - PIV) and temperature measurement (by T-type thermocouples) in an insulated box equipped with a Phase Change Material (PCM). The study was conducted in an empty box and a loaded box with extruded polystyrene slabs (XPS) and methylcellulose slabs (test product). The measurement was conducted at the middle plane and lateral plane. This article contains a complete dataset along with the illustrated figures of conducted experiment. They lead to more understanding of phenomena inside a closed cavity with a cold source and can be useful for validating numerical models, e.g., the results computed by computational fluid dynamic.

**Specifications Table**

**Table:** 

Subject	Bioengineering
Specific subject area	Air velocity and temperature fields measured in an empty and loaded insulated box with a cold source generated by Phase Change Material
Type of data	Table Image Graph MATLAB code Dataset
How the data were acquired	Air velocity measured by Particle Imagery Velocimetry (PIV) Temperature measured by calibrated thermocouples T-Type.
Data format	Raw Analyzed
Description of data collection	Air velocity (at the middle plane and lateral plane of YZ plane and XZ plane) and temperature (at the middle plane and lateral plane of YZ plane and half of the middle plane of XZ plane) in insulated boxes equipped with Phase Change Material (PCM) under following factors: PCM position (on a side and at the top) and Loading condition (no load, loaded with XPS and loaded with test product)
Data source location	INRAE (FRISE Research unit) Antony France
Data accessibility	With the article Repository name: Mendeley Data Data identification number: 10.17632/r7gj93tvz9.1 10.17632/yjbpwtx3c4.1 Direct URL to data: https://data.mendeley.com/datasets/r7gj93tvz9/1 https://data.mendeley.com/datasets/yjbpwtx3c4/1 Repository name: GitHub Data identification number: 10.5281/zenodo.6900688 Direct URL to data: https://github.com/Tanathepl/Temperature-contour.git.
Related research article	Leungtongkum, T., Laguerre, O., Flick, D., Denis, A., Duret, S., & Chaomuang, N. (2022). Experimental investigation of airflow and heat transfer by natural convection in an insulated box with a Phase Change Material using a Particle Image Velocimetry technique. J. of Food Engineering, 111207. https://doi.org/10.1016/j.jfoodeng.2022.111207

## Value of the Data

• The experimental data of air velocity, temperatures of air, and product enable an understanding of the relation between heat transfer and airflow by natural convection in a closed cavity with a cold source.
• The different loading conditions (no load, load with extruded polystyrene, and load with test product) allow the understanding of the impact of loading and the heat exchange between the load and the internal air.
• These data are rare in literature for food transport in an insulated box because the methodology of low air velocity measurement is complicated, i.e., appropriate tracer, laser lighting power, and the time interval between image acquisitions.
• Data can be used/reused to compare with the CFD (Computational Fluid Dynamic) simulation results.

## 1 Data Description

Data presented in this article include raw data of air velocity and temperature measurement, figures of air velocity field and temperature contour field in an insulated box equipped with a Phase Change Material (PCM) under various PCM position and loading condition. Table 1 describes all experimental conditions, only the corresponding figures of the conditions 1 to 4 are presented in this data paper, the ones of the conditions 5 and 6 can be found in Leungtongkum et. al. [1].

**Table 1:** Experimental conditions.

Condition	PCM position	Loading condition	Corresponding figures
1	Side wall	No load	1 to 3
2	Top	No load	4 to 6
3	Side wall	XPS (4 slabs)	7 to 10
4	Top	XPS (4 slabs)	11 to 14
5	Side wall	Test product (16 packs)	10 and 12 of Leungtongkum et. al. [1]
6	Top	Test product (16 packs)	13 to 14 of Leungtongkum et. al. [1]

XPS = extruded polystyrene slabs

The air velocity component, its magnitude and uncertainty in the insulated box under these conditions are shown in Dataset 1: Experimental investigation in an insulated box (Air velocity) (https://data.mendeley.com/datasets/yjbpwtx3c4/1)

The average temperature in the insulated box under these conditions are shown in Dataset 2: Experimental investigation in an insulated box (Average temperature) (https://data.mendeley.com/datasets/r7gj93tvz9/1)

The temperature contour field was drawn via MATLAB by interpolating the measured temperature at different positions during stable conditions. The codes of this drawing are shown in Tanathepl/Temperature-contour (https://github.com/Tanathepl/Temperature-contour.git.)

**Fig. 1:**
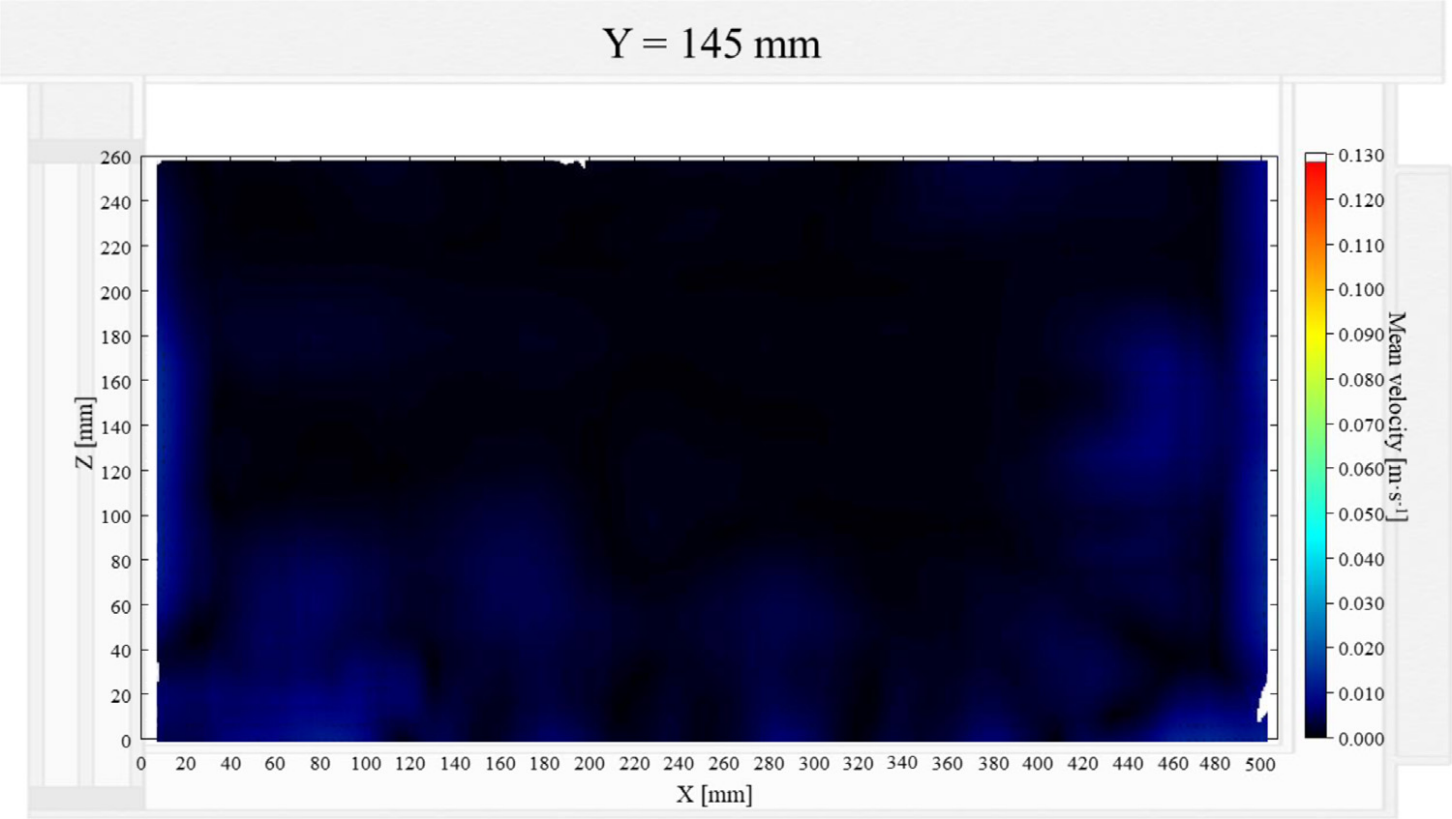
Air velocity field at Y = 145 mm in an unloaded horizontal box with PCM on a side.

**Fig. 2:**
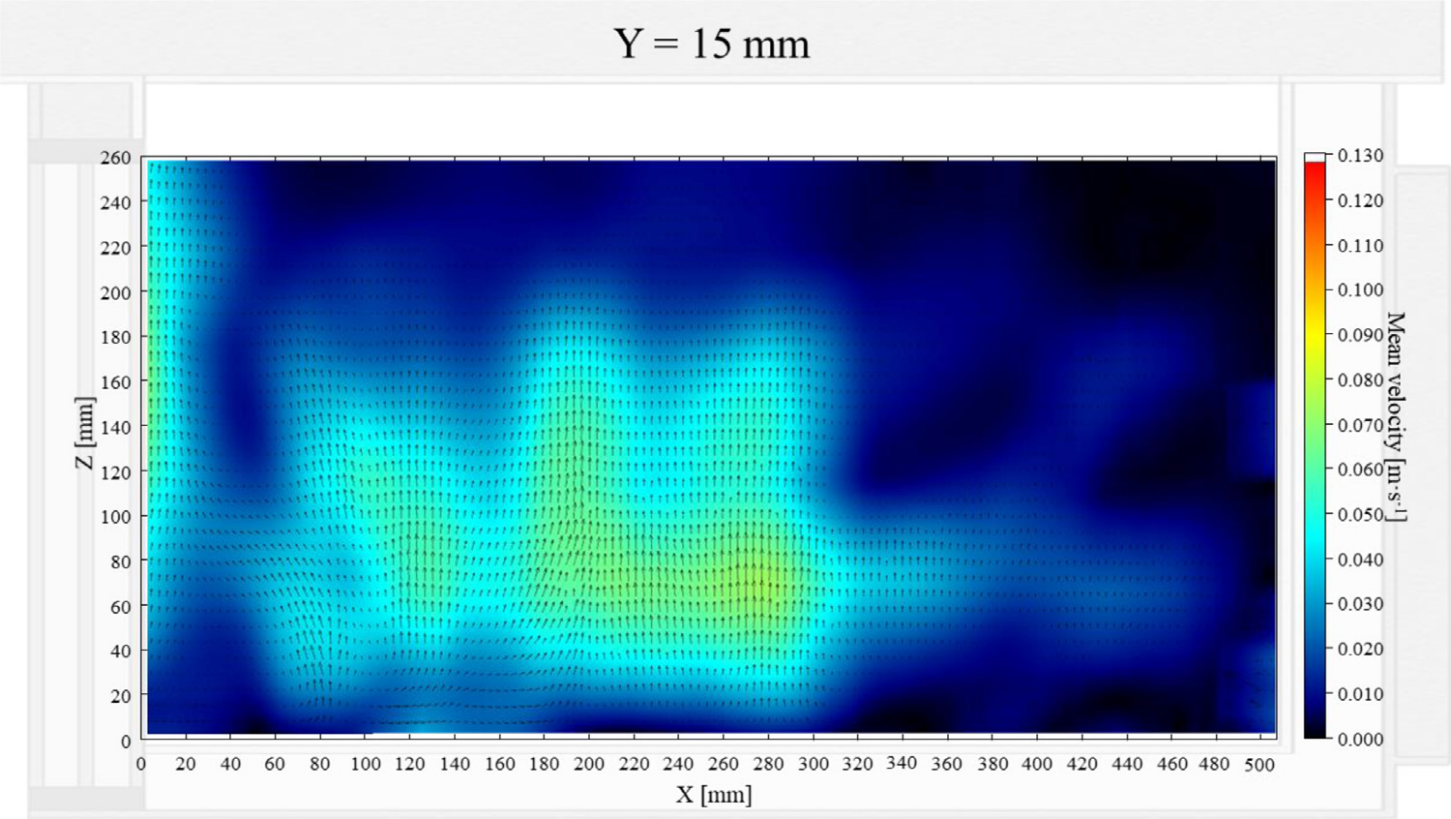
Air velocity field at Y = 15 mm in an unloaded horizontal box with PCM on a side.

**Fig. 3:**
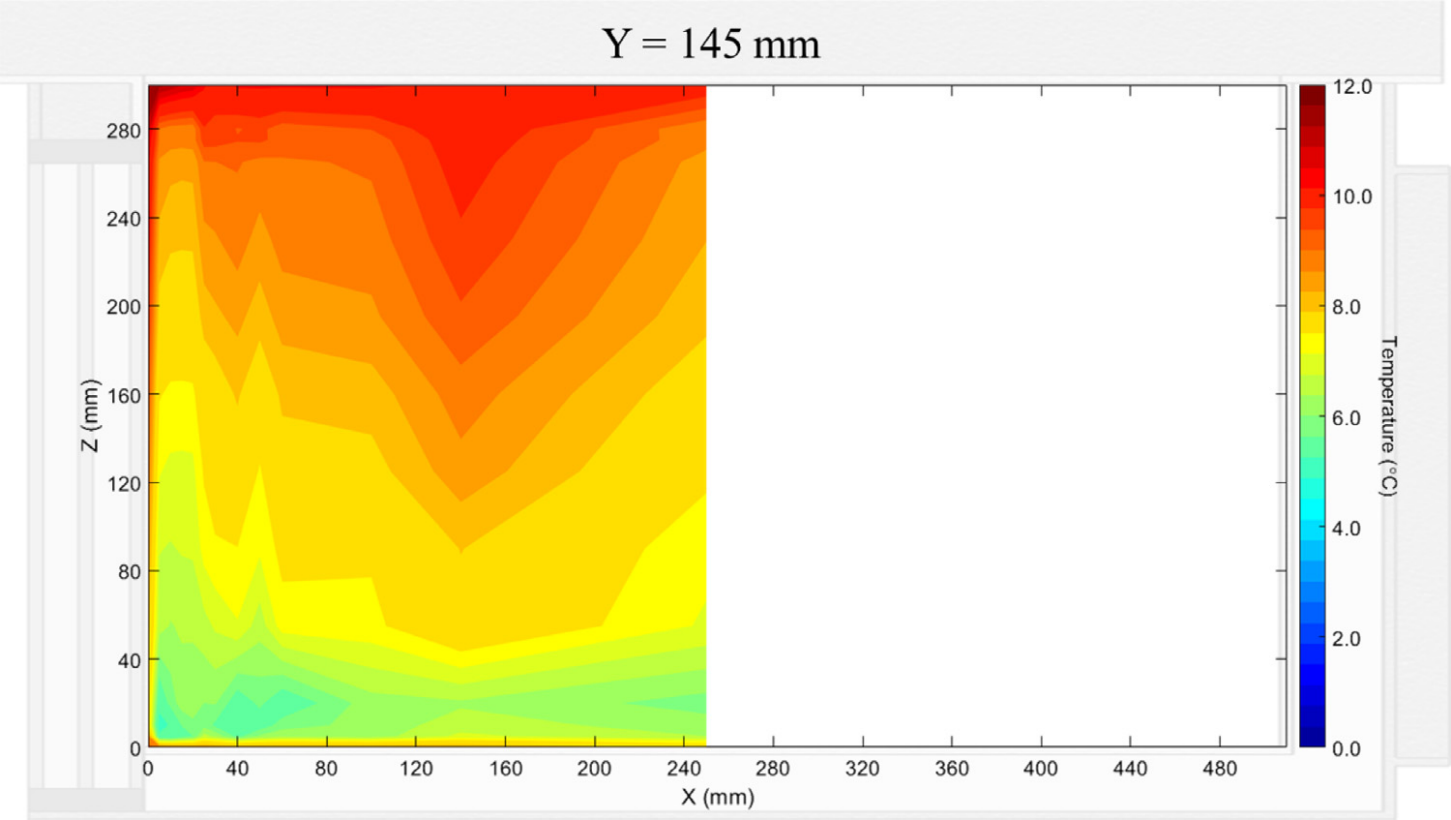
Temperature field at Y = 145 mm in an unloaded horizontal box with PCM on a side.

The air velocity field at Y = 145 mm and 15 mm in the unloaded box with PCM on a side are shown in Figs. 1 and 2. The temperature field at Y = 145 mm is shown in Fig. 3, due to the symmetry of this plane, only a half of the results is shown. Air velocity field at X = 250 mm and 15 mm and temperature field at X = 250 mm are shown in Figs. 5a, b and 6a of Leungtongkum et.al [1].

**Fig. 4:**
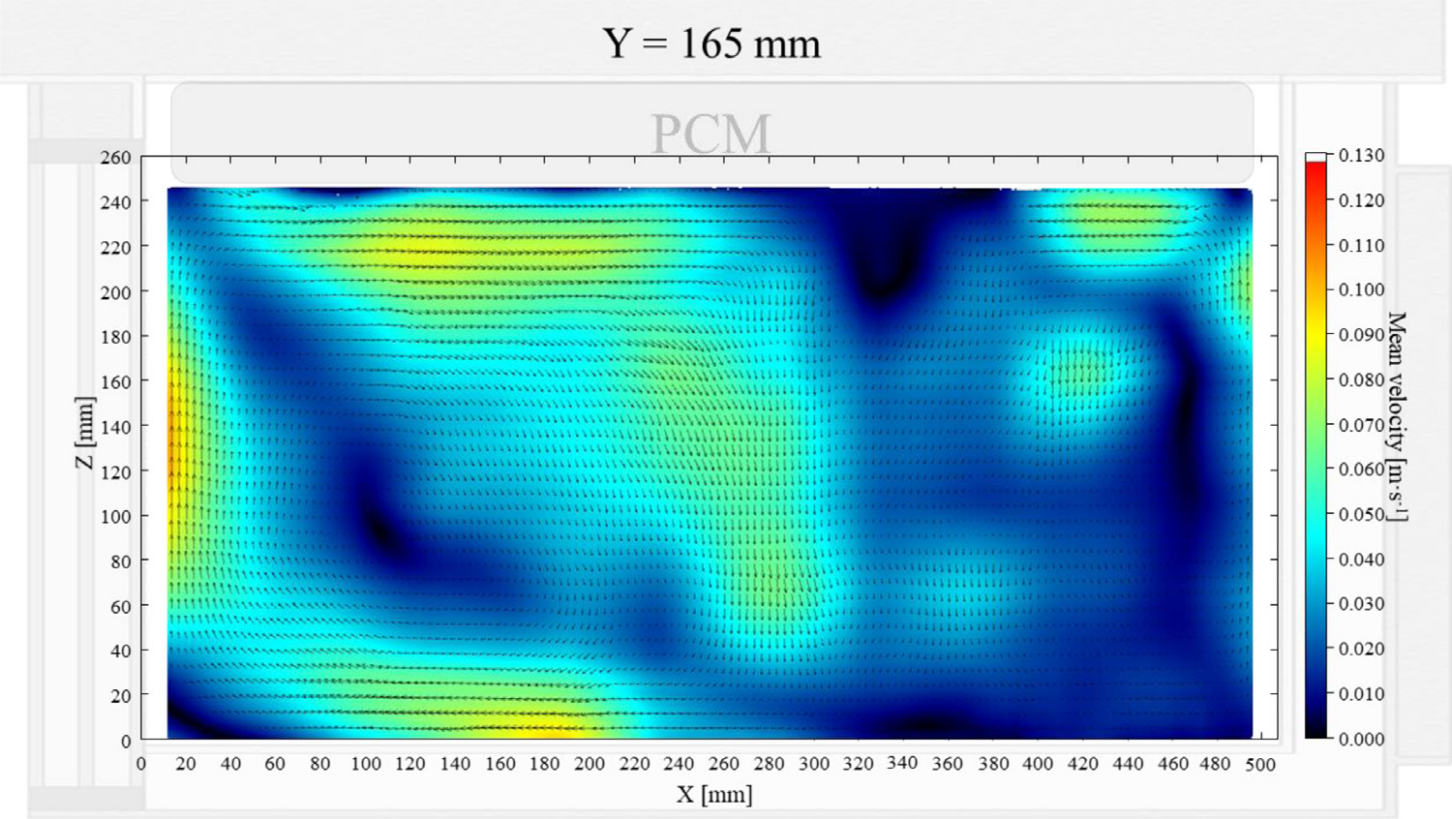
Air velocity field at Y = 165 mm in an unloaded horizontal box with PCM at top.

**Fig. 5:**
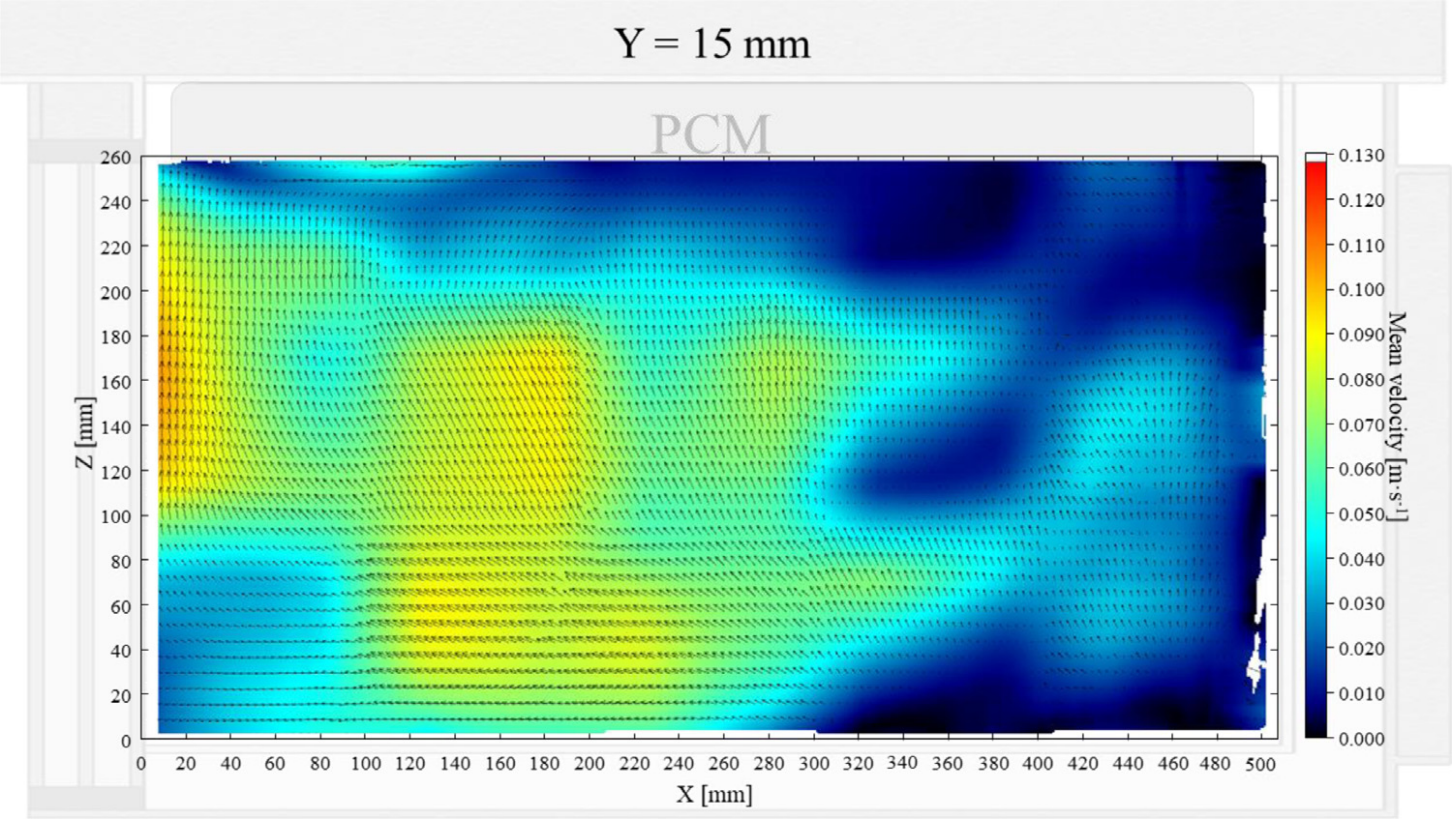
Air velocity field at Y = 15 mm in an unloaded horizontal box with PCM at top.

**Fig. 6:**
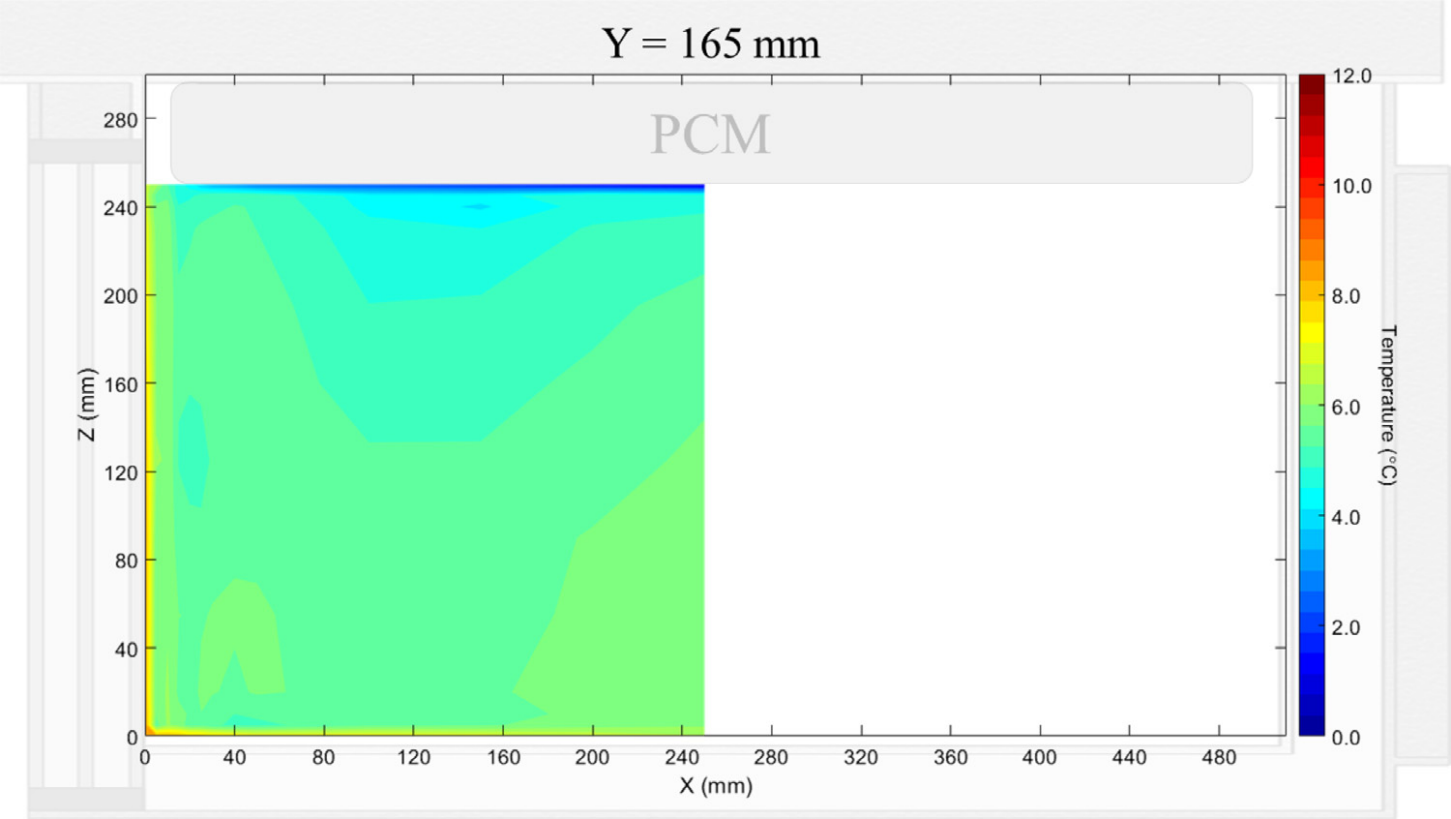
Temperature field at Y = 165 mm in an unloaded horizontal box with PCM at top.

**Fig. 7:**
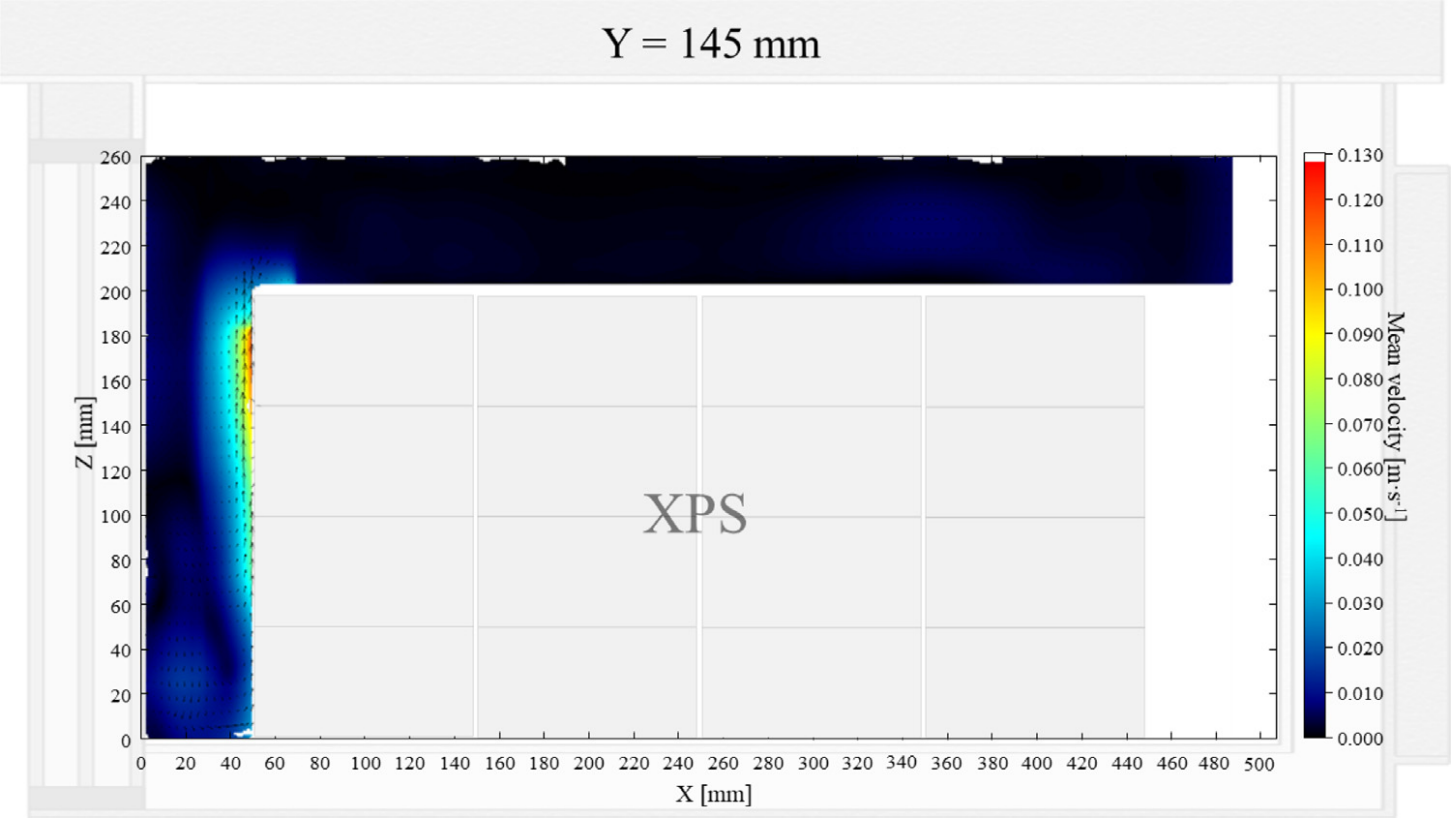
Air velocity field at Y = 145 mm in a box loaded with XPS and PCM on a side. White area on the right represents the unmeasurable zone because of the inaccessibility of laser sheet.

Air velocity field at Y = 165 mm and 15 mm and temperature field at Y = 165 mm (half of the plane) in an unloaded box with PCM at top were shown in Fig. 4, Fig. 5, Fig. 6. Air velocity field at X = 250 mm and 15 mm and temperature field at X = 250 mm are shown and discussed in Figs. 7a, b and 8a of Leungtongkum et al. [1].

**Fig. 8:**
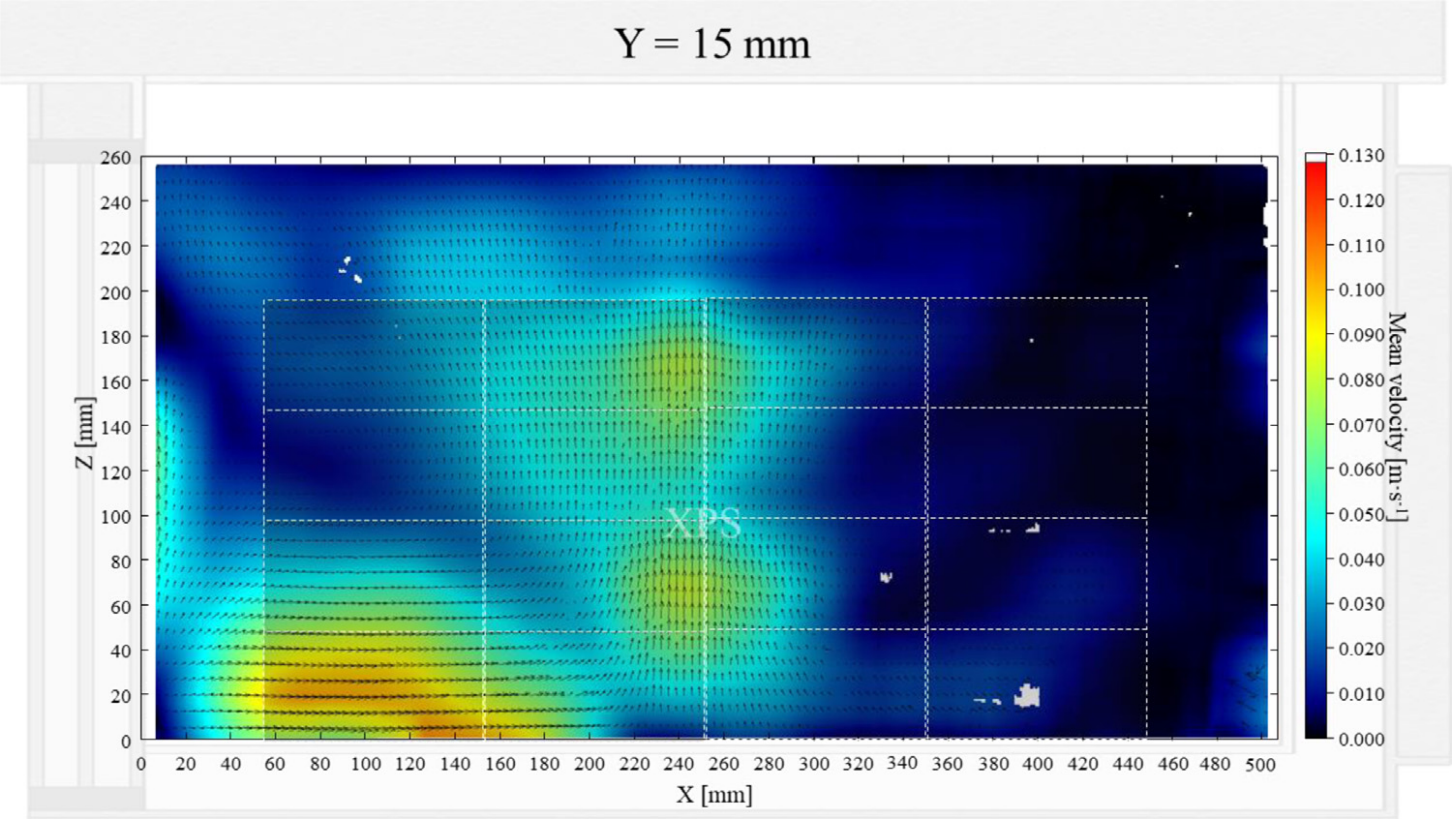
Air velocity field at Y = 15 mm in a box loaded with XPS and PCM on a side.

**Fig. 9:**
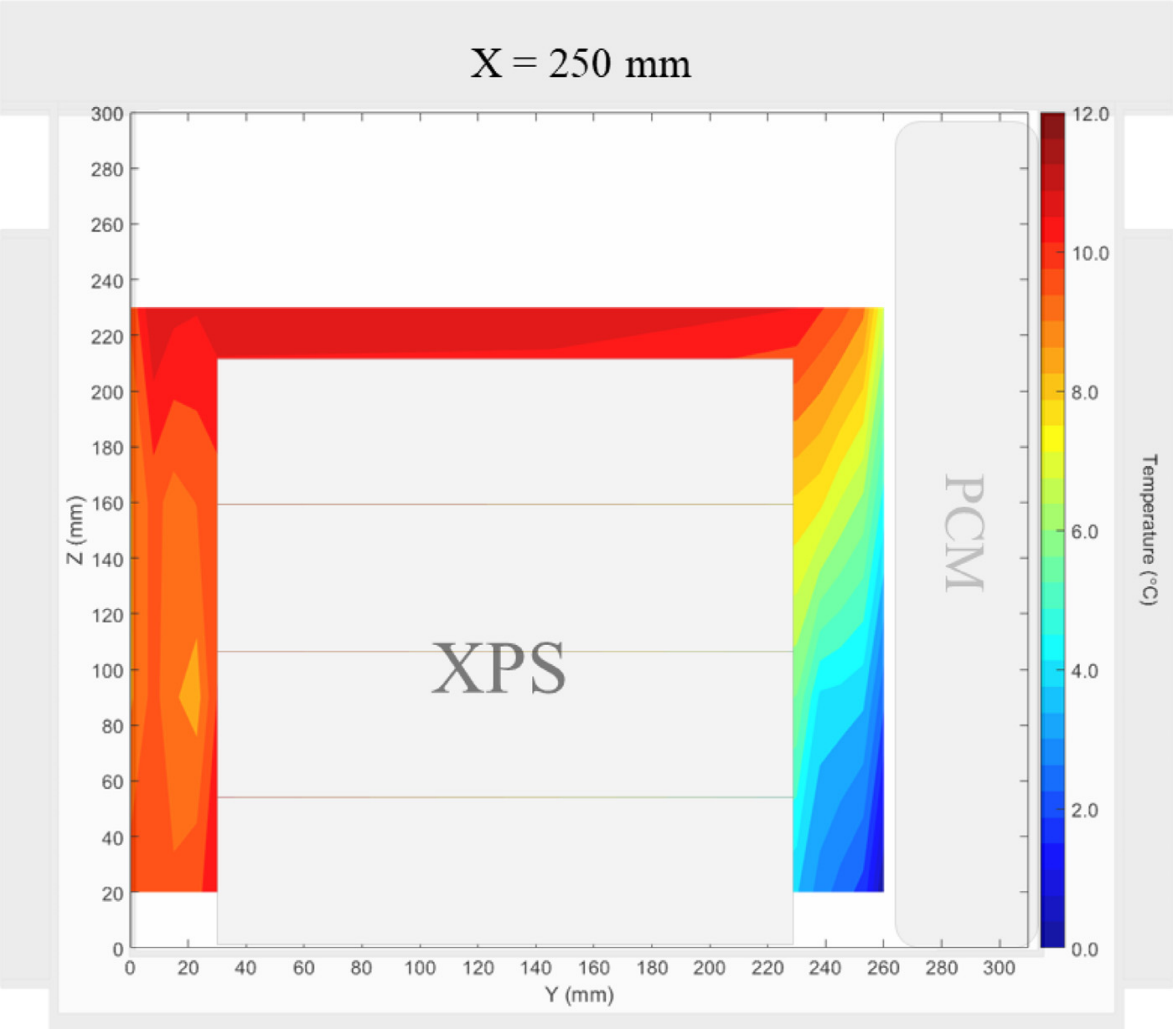
Temperature field at X = 250 mm in a box loaded with XPS and PCM on a side.

**Fig. 10:**
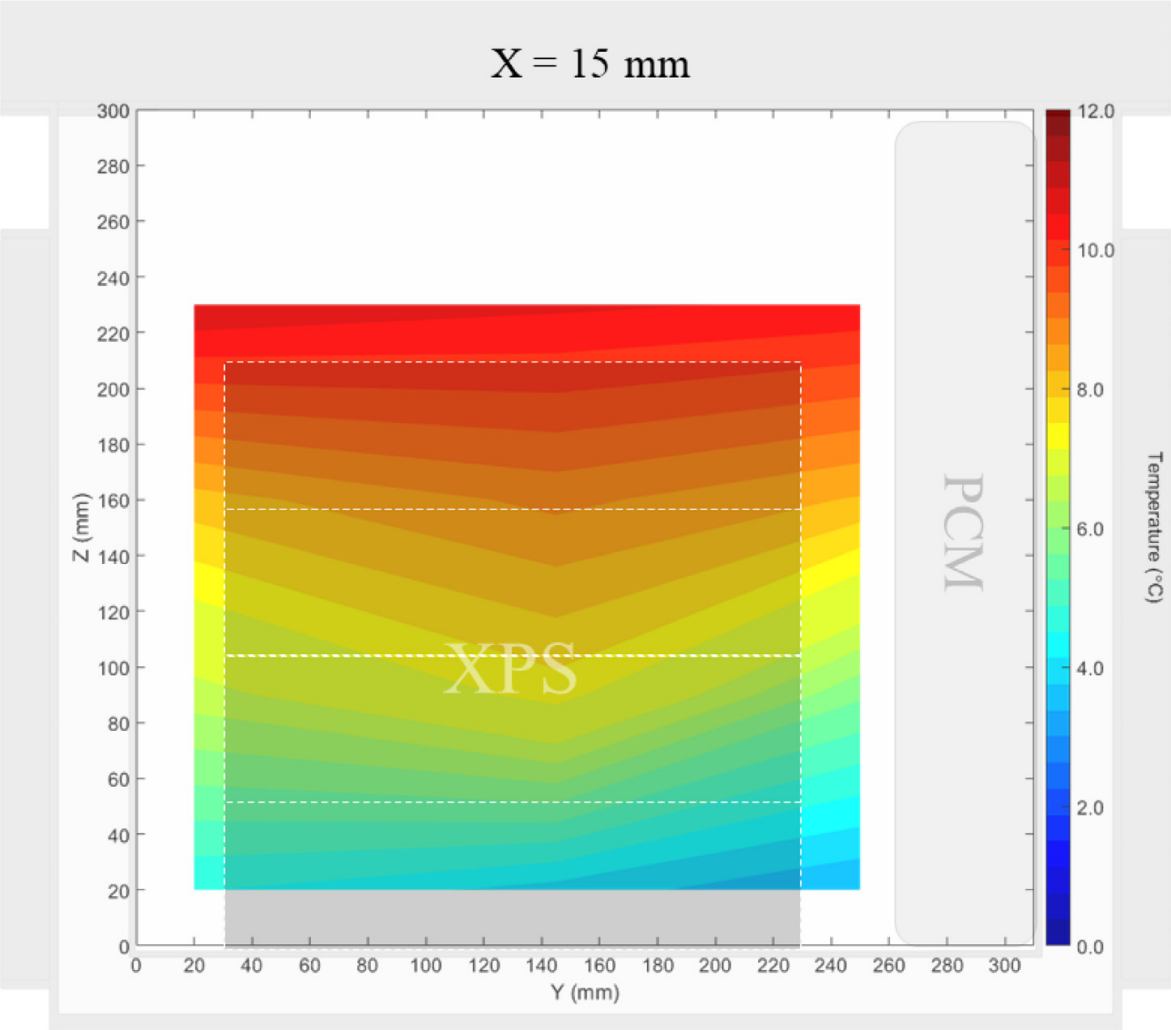
Temperature field at X = 15 mm in a box loaded with XPS and PCM on a side.

Air velocity field at Y = 145 mm and 15 mm and temperature field at X = 250 mm and X = 15 mm in a box loaded with XPS and PCM on a side were shown in Fig. 7, Fig. 8, Fig. 9, Fig. 10. Air velocity field for X = 250 mm and 15 mm are shown and discussed in Fig. 10a and b of Leungtongkum et.al [1].

**Fig. 11:**
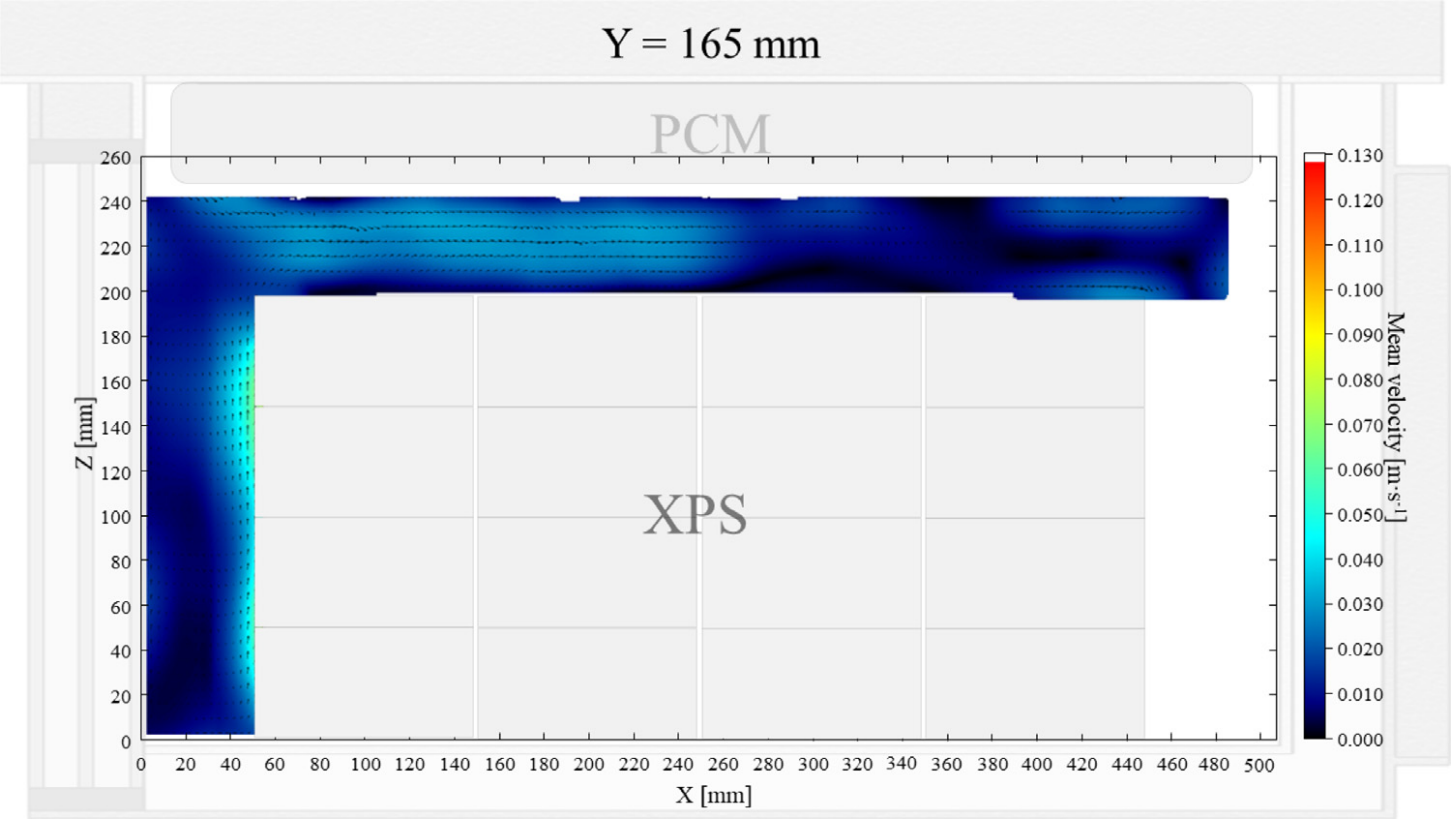
Air velocity field at Y = 165 mm in a box loaded with XPS and PCM at top. White area on the right represents the unmeasurable zone because of the inaccessibility of laser sheet.

**Fig. 12:**
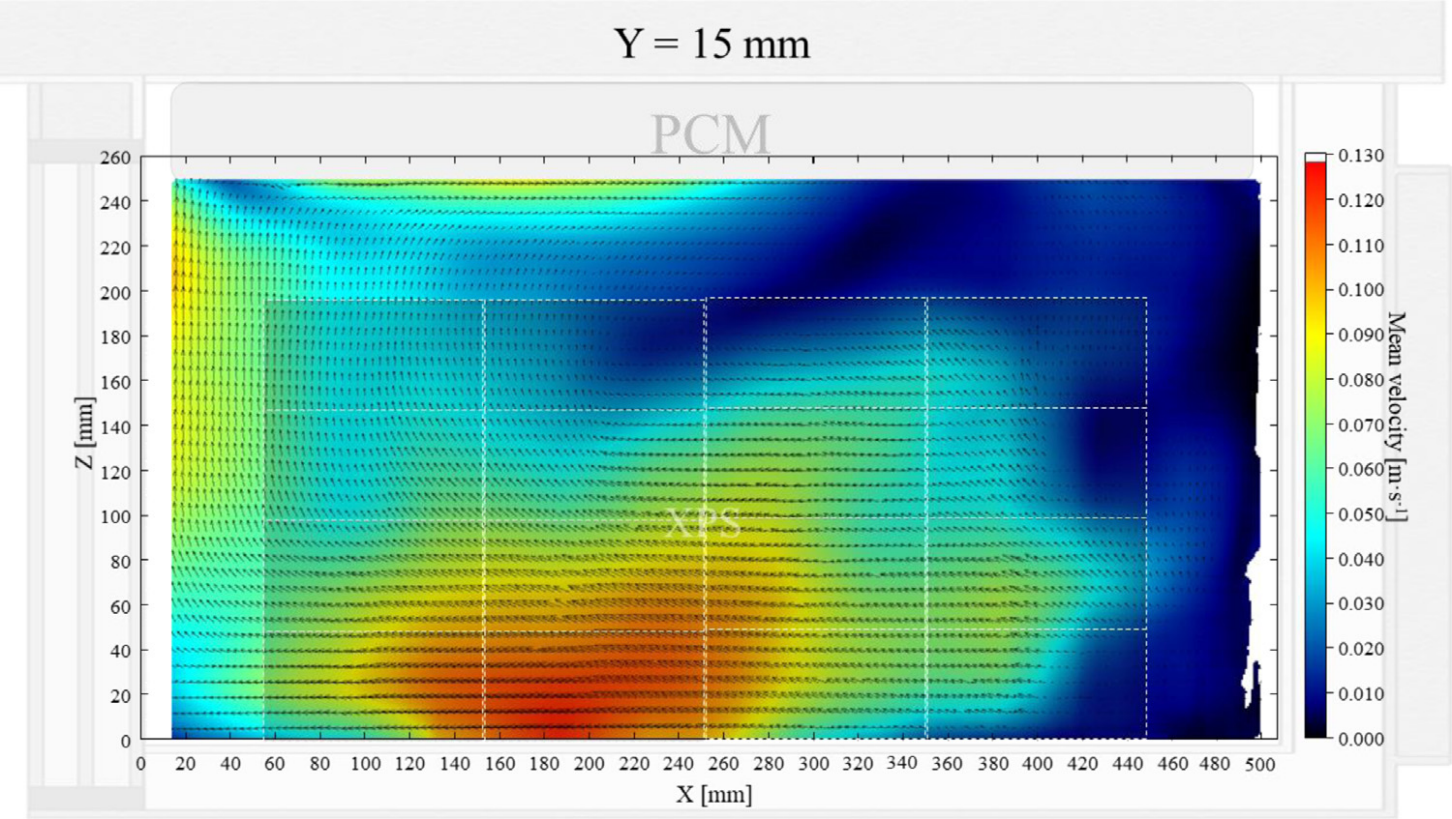
Air velocity field at Y = 15 mm in a box loaded with XPS and PCM at top.

**Fig. 13:**
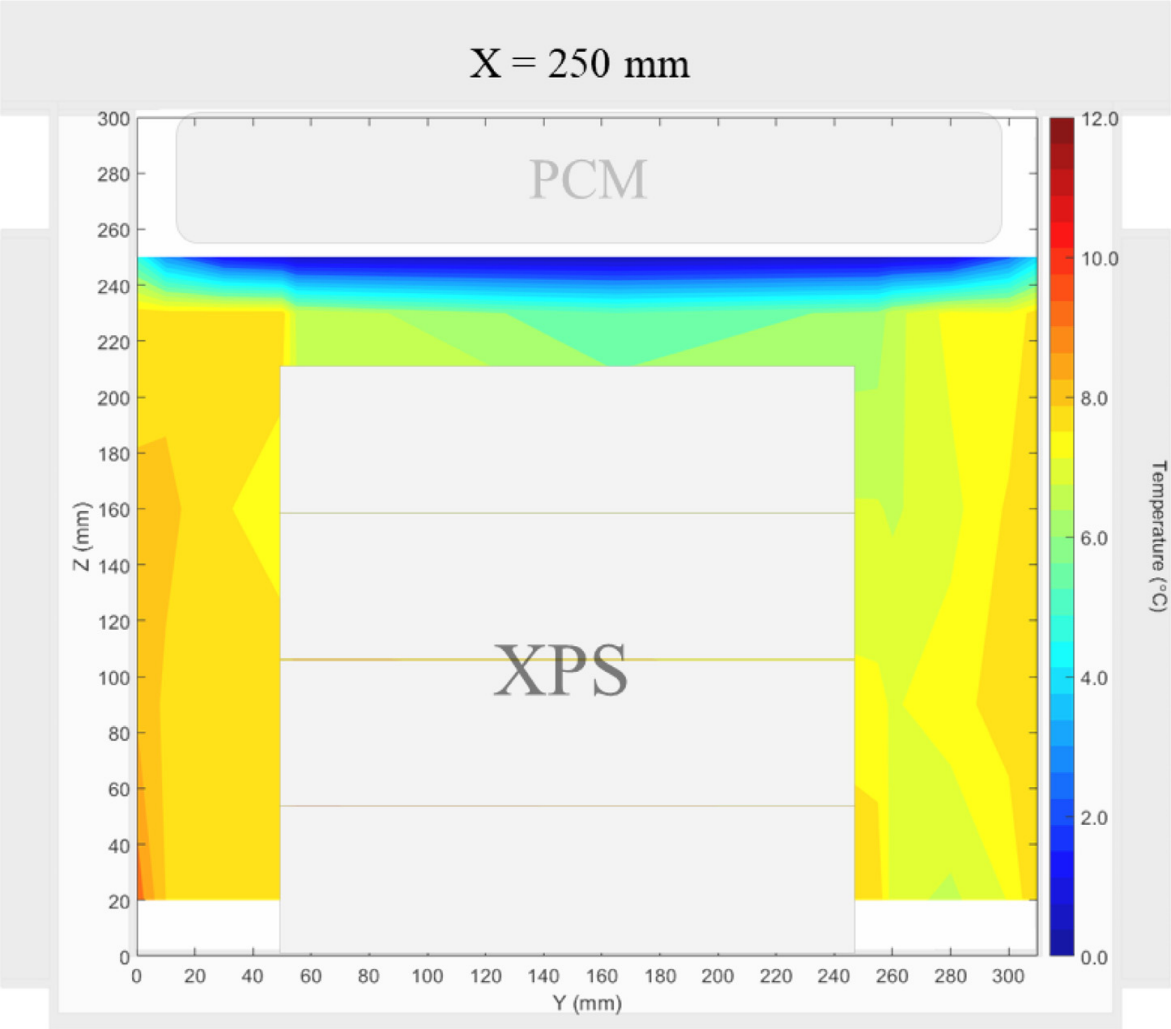
Temperature field at X = 250 mm in a box loaded with XPS and PCM at top.

**Fig. 14:**
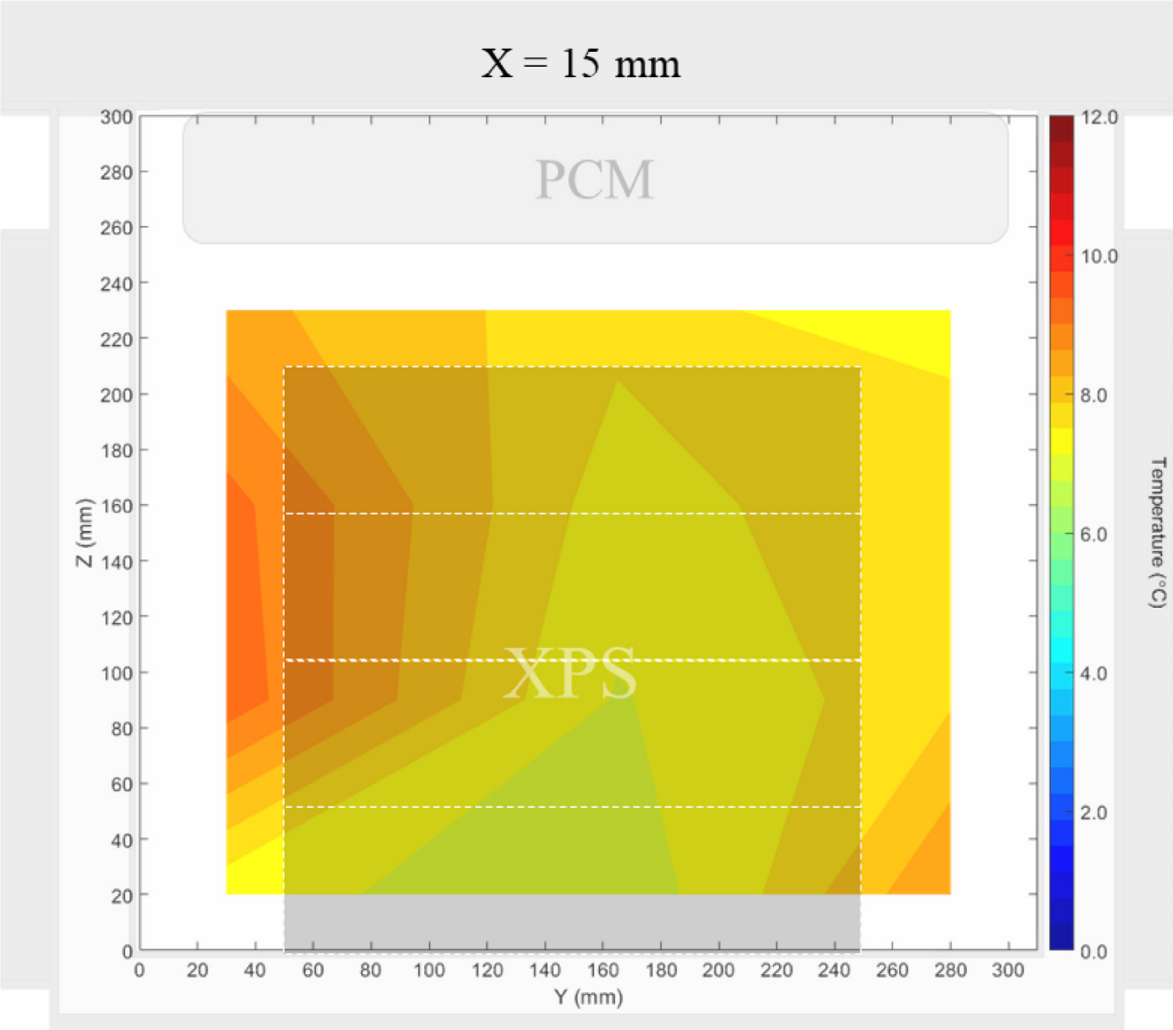
Temperature field at X = 15 mm in a box loaded with XPS and PCM at top.

Air velocity field at Y = 165 mm and 15 mm and temperature field at X = 250 mm and X = 15 mm in a box loaded with XPS and PCM at top were shown in Fig. 11, Fig. 12, Fig. 13, Fig. 14. Air velocity field for X = 250 mm and 15 mm are shown and discussed in Fig. 13a and b of Leungtongkum et.al [1].

## 2 Experimental Design, Materials and Methods

### 2.1 Material

**Fig. 15:**
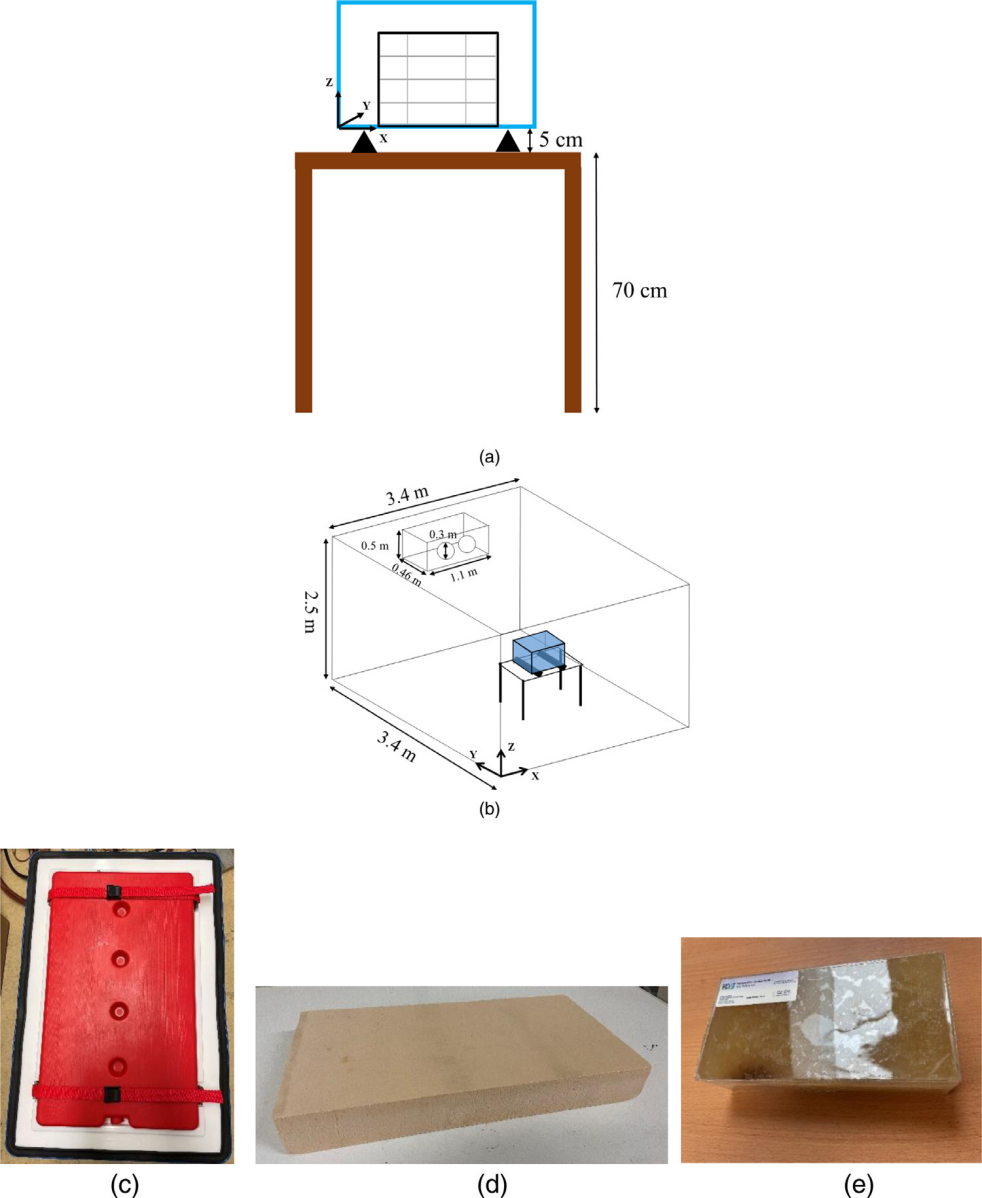
Materials and experimental setup (a) the box with wood support on the table, (b) experimental setup, (c) PCM container, (d) extruded polystyrene slab, and (e) test product pack.

Fig. 15 shows the materials and experimental setup. Two boxes were used, for the thermal study, the commercialized multilayer insulated box (Manutan SA, Gonesse, France) with 500 mm × 310 mm × 300 mm internal dimensions and 570 mm × 380 mm × 370 mm external dimensions is used. The side and bottom walls contain expanded polystyrene (25 mm thickness), polypropylene (inner and outer layers of 3.5 mm thickness), and an air gap is expected between the expanded polystyrene and the inner layer (estimated thickness: 5 mm). The other box is for the airflow study and has the same dimensions and wall structure, but two side walls are replaced by triple-glazed windows (3 glass panes, each with a thickness of 4 mm, two argon-filled 10-mm gaps). The illustration of these two boxes is shown in Fig. 1 of Leungtongkum et al. [1]. The box was positioned on a 50-mm height wooden support to ensure homogeneous heat exchange with the ambient air around the box (Fig. 15a). This box was placed on a 0.7-m height table in the center of the 3.4 m × 3.4 m × 2.5 m temperature-controlled test room. The PCM container (Fig. 15c), made of polypropylene (2.5-mm thickness) and filled with 3.5 kg of tap water (melting point ∼ 0°C), has external dimensions of 460 mm × 280 mm × 50 mm. The extruded polystyrene slab (XPS) has a dimension of 400 mm × 200 mm × 50 mm (Fig. 15d). The test products (with 200 mm × 100 mm × 50 mm pack dimensions – Fig. 15e) contained 23% methylhydroxyethylcellulose, 76.4% water, and 0.5% NaCl (Refrigeration Development and Testing Ltd., North Somerset, UK) Table 2 shows thermal conductivity at 300 K of each material.

**Table 2:** Thermal conductivity at 300 K of each material.

Material	k (W m^−1^ K^−1^)	Reference
Polypropylene	0.12	[2]
Expanded polystyrene	0.029	[2]
Air	0.026	[3]
Argon	0.018	[4]
Glass	1.4	[3]

Thermal conductivity (k) of each box wall was determined by calculation using thermal conductivity and thickness of each layer as in eq. 1

(1) $$k=\;\frac{1}{\sum_{i=1}^{n}\frac{x_{i}}{k_{i}}}$$

Where k is thermal conductivity of material (W m^−1^ K^−1^) and x is thickness (m)

Thus, the thermal conductivity of the insulated wall and the glass-pane wall is 0.90 W m^−1^ K^−1^ and 0.89 W m^−1^ K^−1^, respectively.

The experiment using the internal heating method (adapted from ATP [5]) was also conducted to measure the heat transmission coefficient (K) with 20 W and 30 W heating. Firstly, 14 thermocouples were placed inside the box (8 at 50 mm from each corner and 6 at 50 mm from the center of each surface). The other 14 were put outside the box in the same manner. The heating unit consisted of adjustable resistance and a fan. After setting the heating power, the system was left until reaching a steady state (temperature fluctuation is less than ±0.3°C for 12 h with less than ±1.0°C during the preceding 6 h.

Data over at least 6 h was averaged to get the mean temperature at each position. Next, mean data of either inside or outside the box was averaged to get internal and external temperature, respectively. The heat transmission coefficient can be calculated by eq.2

(2) $$K=\;\frac{\dot{Q}}{A\Delta T}$$

Where K is heat transmission coefficient (W m^−2^ K^−1^)

$\dot{Q}$ is heating power (W)
$A=\;\sqrt{A_{int}A_{ext}}$ is mean surface area between internal surface and external surface (m^2^)
$\Delta T=\;T_{in}-\;T_{out}$ is temperature difference between inside and outside of the box (K)

The experimental value of heat transmission coefficient is shown in Table 3.

**Table 3:** Experimental value of heat transmission coefficient.

Box type	Power (W)	A (m^2^)	T_in_ (°C)	T_out_ (°C)	K (W m^−2^ K^−1^)
Box A (Insulated box)	20	1.027	37.52	5.53	0.61
	30	1.027	46.76	5.44	0.71
Box B					
(Glass-pane box)	20	1.027	36.21	6.13	0.65
	30	1.027	43.9	6.22	0.78

### 2.2 Thermal study

#### 2.2.1 Experimental setup

The temperature was measured by T-type thermocouples linked to the Agilent 34972A data acquisition unit (Agilent Technologies, CA, USA). These thermocouples were calibrated at -10°C, 0°C, 10°C, 20°C and 30°C with a precision of ±0.2°C.

PCM slab was placed horizontally in a freezer set at -2°C for at least 48 h before each experiment.

Sixteen packs of test product were put in a polystyrene box and stored in a domestic refrigerator set at 4°C or 10°C for at least 24 h before each experiment to assure the homogeneous product's initial temperature.

#### 2.2.2 Temperature measurement in an unloaded box

Firstly, all thermocouples were placed in the box. Air temperature distribution was measured at the middle plane of the YZ plane (X = 250 mm) with various y and z positions and around half of the XZ plane (Y = 145 mm for the box with PCM on the side and Y = 165 mm for that with PCM at the top). Two stands, each equipped with 12 thermocouples spreading over the height (z-axis), were displaced inside the box in x and y direction to obtain the air temperature field. Thermocouples were also put inside the PCM (at half thickness), on the surface of the PCM container, and the box's internal walls. The diagram showing thermocouples positions is in Fig. 2a of Leungtongkum et al. [1].

Ninety minutes after the box was closed (when the steady state was reached), temperatures were recorded for at least 5 min. (acquisition interval 15 s). Then, the box was opened in order to move the stands prior to closing the box again. This process took less than 1 min. to avoid disturbances caused by external air to the greatest possible extent. Fifteen minutes later (when the steady state was reached again), temperatures were recorded over 5 min. before re-opening the box. Then, the position of the stands was changed in y direction (18 to 21 positions), allowing 200 measurement points in total. A temperature contour map was plotted by MATLAB with interpolation from these measurements.

**Fig. 16:**
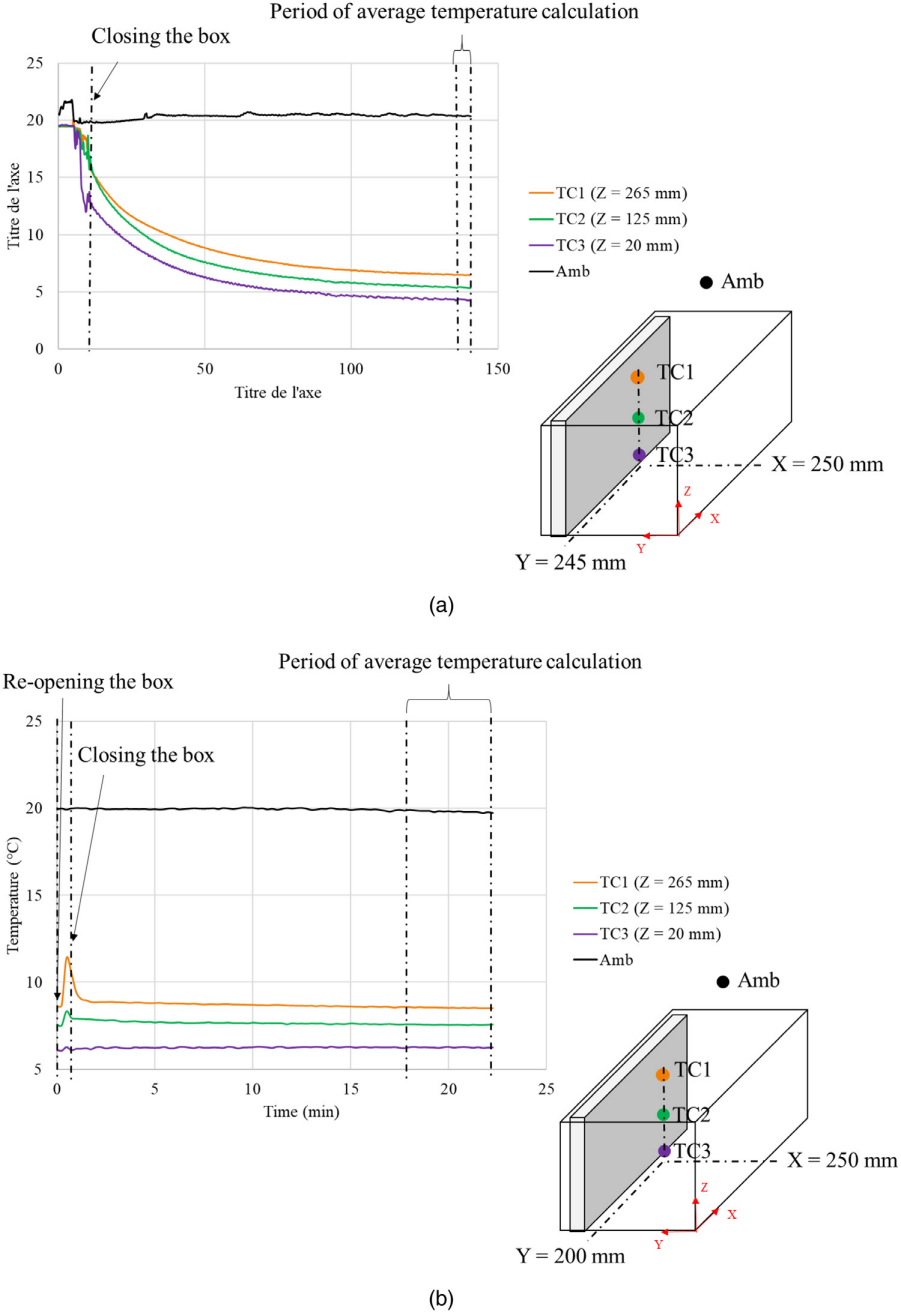
Temperature evolution of some locations in an unloaded horizontal box with PCM on a side under 20°C ambient) of (a) the first measurement and (b) the following measurement.

Fig. 16 illustrates the temperature evolution of some locations in an unloaded horizontal box with PCM on a side under 20°C ambient during the first measurement (Fig. 16a) and the following measurement (Fig. 16b). These figures show the period during which the average temperature was calculated: 135 min. to 140 min. after box closing for the first measurement and 17 min. to 22 min. for the following measurement. Then, these average temperatures were used to present the temperature field in stable condition in the following figures (Figs. 1, 2, 4, and 5).

#### 2.2.3 Temperature measurement in a loaded box

For loaded boxes, it was loaded with four slabs of XPS or 16 test product packs. The temperature measurement was carried out at fixed positions with 29 thermocouples on the surface of the XPS slab, and the test product and core of the test product located at the middle plane (x = 250 mm) and with ten thermocouples at the lateral plane (x = 15 mm). The diagram showing thermocouples positions is in Fig. 2b of Leungtongkum et al. [1]. The measurement started after the box closing until complete PCM melted without the box opening during the experiment. The temperatures at the middle plane (x = 250 mm) were recorded continuously (every 30 s). The results of the period ranging from 400 min. to 600 min. were analyzed and compared. The temperature contour map was drawn by MATLAB with interpolation.

**Fig. 17:**
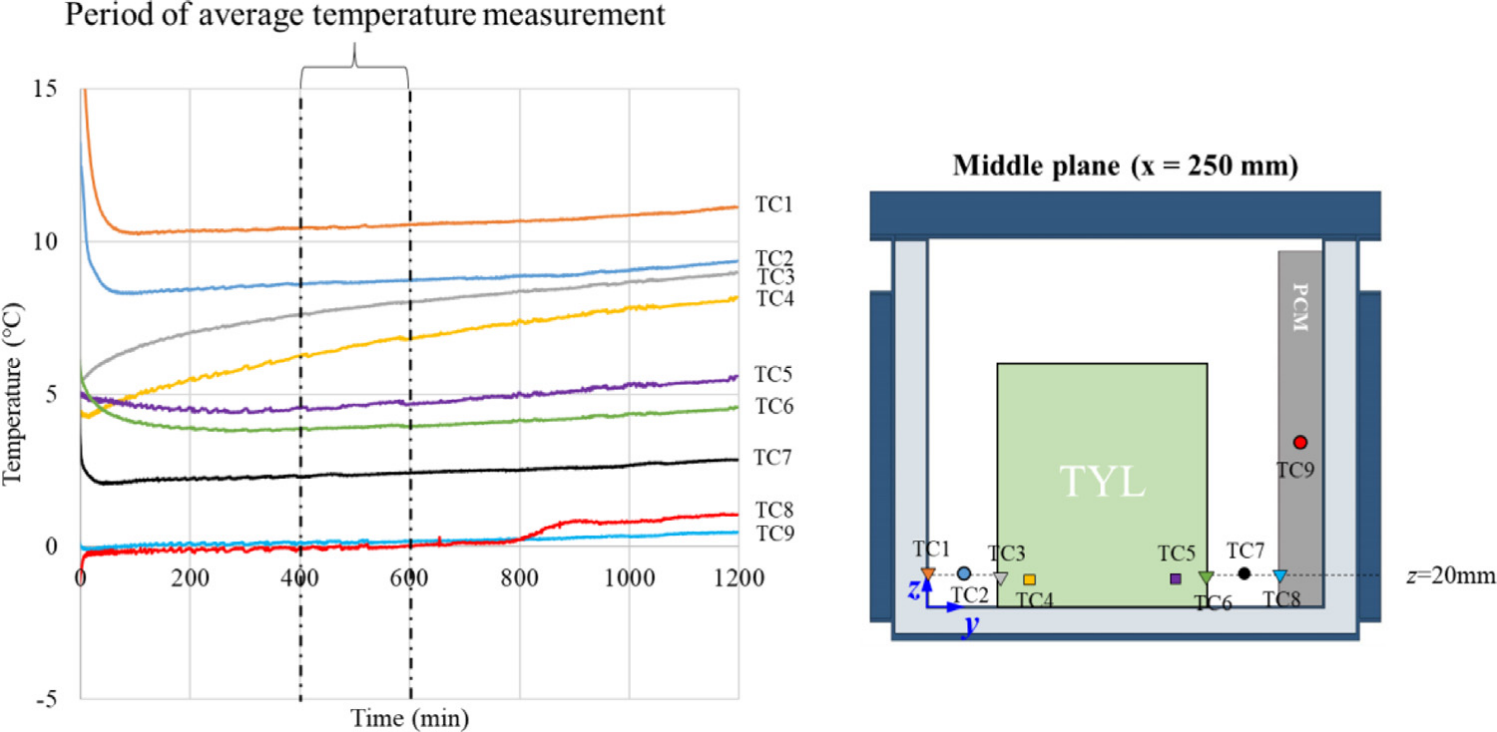
Temperature evolution at the bottom of a loaded horizontal box with PCM on a side loaded with test product.

Fig. 17 shows temperature evolution at the bottom of a loaded horizontal box with PCM on a side under 20°C ambient, product initial temperature = 4°C. This figure shows the period from 400 min. to 600 min. during which the average temperature was calculated. Then, these average temperatures were used to present the temperature field in stable condition in the following figures (Figs. 7, 8, 11, and 12).

### 2.3 Airflow study

#### 2.3.1 Instrumentation

The oil-based smoke tracer with a 0.3 μm mean diameter was precooled in a container containing four PCM packs, reducing the smoke temperature from about 50°C to around 10°C. A small fan guided smoke to the box through a connecting duct while a valve controlled its flow rate.

A 2D-PIV system (LaVision, FlowMaster 2D) comprised three main components: a double-pulsed Nd:YLF laser (527 nm wavelength, 10 mJ pulse energy), a high-speed 12-bit CMOS video camera (Photron, FASTCAM SA3; 1024 × 1024 pixels in resolution) connected with a lens (Sigma; 105 mm, f/1:2.8) and a programmable timing unit (PTU-X) for the synchronization of laser and camera. Smoke particle scattering during laser pulses visualized the airflow pattern inside the cavity.

The camera and the laser were mounted on a three-dimensional displacement system (precision of displacement ±1 mm). This system makes the perpendicularity between the camera view and the light sheet (1-mm thickness) possible. The diagram and photograph of the experimental setup for the airflow study are shown in Fig. 3 of Leungtongkum et al. [1].

#### 2.3.2 Image acquisition

For each measured window, 500 pairs of images were recorded every 20 ms with a time interval of 900 μs between two images of the same pair, in other words, between two pulsed laser illuminations. The total duration of measurement was 10 s.

#### 2.3.3 Image post-processing

A multi-pass correlation algorithm was applied to calculate the instantaneous airflow vector. The cross-correlation between each image in the pair was accomplished by decreasing interrogation window sizes: 64 × 64 pixels with 50% overlap for the first pass and 32 × 32 pixels with 75% overlap for the three following passes. In each pass, all interrogation windows at the same position on the paired images were cross-correlated and independently produced a single displacement vector, then were later merged to produce a 2D vector field of the whole image. After 500 instantaneous vector fields had been attained, the mean velocity field of each measured window was calculated by Eq. 3.

(3) $$\bar{v}=\frac{1}{N}\sum_{i=1}^{N}\sqrt{v_{y,i}^{2}+v_{z,i}^{2}}$$

where N is the total number of measured images of the same windows (N = 500 in our study) and v_y_ and v_z_ are the horizontal and vertical velocity components in m s^−1^, respectively.

The mean velocity fields of all measured windows were then combined to construct the velocity field of the entire measurement plane. It should be highlighted that the out-of-plane regions and the regions near the reflection surface in the images (e.g., the surfaces of PCM, walls, and the load) were excluded prior to the vector calculation.

The DaVis software employs the correlation statistics method to estimate the uncertainty of the PIV measurement. As discussed in Wieneke [6], the uncertainty is quantified based on the statistical analysis of the correlation process using differences in the intensity pattern of the two images.

#### 2.3.4 Experimental protocol

Air velocity measurements were conducted under stable conditions: after 90 min. for unloaded boxes, and after 4 hours for loaded boxes. The PIV measurement was started 30 min. afterward. It is to be emphasized that the standard deviations of the air temperatures at different positions in the box did not exceed 0.3°C during PIV measurement.

The PIV measurements were performed on the middle plane of the YZ plane (x = 250 mm), on the lateral plane of the YZ plane (x = 15 mm), on the middle plane of the XZ plane (y = 145 mm for the condition with PCM on a side and y = 165 mm for that with PCM at the top) and a lateral plane of XZ plane (y = 15 mm). Based on the image calibration using a ruler and the DaVis software, a magnification factor of 0.108 mm/pixel was defined, and the image size was 115.5 mm × 115.5 mm. The positions of the measured windows were changed by using the displacement system as explained above. Several measured windows with partial overlapping were applied to cover the whole area of the plane.

## Ethics Statement

This work did not involve human subjects, animal experiments and data collected from social media platforms.

## CRediT authorship contribution statement

**Tanathep Leungtongkum:** Conceptualization, Methodology, Investigation, Validation, Formal analysis, Software, Writing – original draft, Visualization. **Onrawee Laguerre:** Validation, Formal analysis, Writing – review & editing, Supervision, Project administration, Funding acquisition. **Denis Flick:** Methodology, Validation, Formal analysis, Writing – review & editing, Supervision. **Alain Denis:** Investigation, Software. **Steven Duret:** Validation, Formal analysis, Writing – review & editing, Supervision. **Nattawut Chaomuang:** Conceptualization, Methodology, Investigation, Validation, Formal analysis, Software, Visualization, Writing – original draft, Funding acquisition.

## Declaration of Competing Interest

The authors declare that they have no known competing financial interests or personal relationships that could have appeared to influence the work reported in this paper.

The authors declare the following financial interests/personal relationships which may be considered as potential competing interests: no known potential competing interests

## Data Availability

Temperature contour (Original data) (GitHub). Experimental investigation in an insulated box (Air velocity) (Original data) (Mendeley Data). Experimental investigation in an insulated box (Average temperature) (Original data) (Mendeley Data)
